# Influence of chemotherapy intensity on opportunistic fungal infection risk in non-small cell lung carcinoma: a retrospective study

**DOI:** 10.3389/fmed.2026.1800575

**Published:** 2026-03-30

**Authors:** Fang Zheng, Wenli Li, Fei Zhao, Zhenzhen Zhu, Weiyang Wang, Qinghai You

**Affiliations:** 1 Department of Respiratory and Critical Care Medicine, First Affiliated Hospital of Anhui Medical University, Hefei, China; 2 Department of Respiratory and Critical Care Medicine, Anhui Provincial Chest Hospital, Hefei, China; 3 Department of Thoracic Oncology, The Fifth Affiliated Hospital of Anhui Medical University, Fuyang, China

**Keywords:** chemotherapy intensity, invasive fungal infection, myelosuppression, non-small cell lung carcinoma, relative dose intensity, retrospective study

## Abstract

**Background:**

Opportunistic fungal infections represent a serious treatment-related complication in patients with advanced non-small cell lung carcinoma (NSCLC) undergoing chemotherapy, closely associated with treatment intensity-induced immunosuppression.

**Objectives:**

To investigate the influence of relative dose intensity (RDI) of first-line platinum-based doublet chemotherapy on the risk of invasive fungal infection (IFI) in patients with stage IIIB-IV NSCLC and its underlying mechanisms.

**Methods:**

A total of 195 patients with stage IIIB-IV NSCLC who received first-line platinum-based doublet chemotherapy between January 2022 and December 2025 were enrolled. Based on the average RDI of chemotherapy, patients were categorized into high-intensity (RDI > 85%, *n* = 68), standard-intensity (RDI 70%–85%, *n* = 74), and low-intensity (RDI < 70%, *n* = 53) groups. Data on the incidence of IFI, febrile neutropenia (FN), infection-related progression-free survival (irPFS), nadir absolute neutrophil count (ANC nadir), duration of profound neutropenia, total infection-related hospitalization days, rate of empirical antifungal use, and overall survival (OS) were retrospectively collected and compared among the three groups.

**Results:**

The incidence of IFI was higher in the high-intensity group than in the low-intensity group (χ^2^ = 15.837, *P* < 0.001). Multivariate Cox regression analysis indicated that high-intensity chemotherapy was independently associated with an increased risk of IFI compared with low-intensity chemotherapy (HR = 8.241, *P* = 0.005). The incidence of FN was higher in the high-intensity group than in the low-intensity group (χ^2^ = 7.892, *P* = 0.019). The duration of infection-related hospitalization was longer in the high-intensity group than in the low-intensity group (H = 19.037, *P* < 0.001). The rate of empirical antifungal use was higher in the high-intensity group than in the low-intensity group (χ^2^ = 13.275, *P* = 0.001). A significant difference in irPFS was observed among the three groups (Log-rank χ^2^ = 11.524, *P* = 0.003), while no significant difference was found in OS (Log-rank χ^2^ = 2.137, *P* = 0.344). Mediation analysis suggested that ANC nadir partially mediated the effect of chemotherapy intensity on IFI risk.

**Conclusion:**

In patients with advanced NSCLC, high relative dose intensity of first-line chemotherapy is independently associated with an increased risk of invasive fungal infection. This association is partly mediated by treatment-induced myelosuppression and is accompanied by a significant increase in clinical burden.

## 1 Introduction

Invasive fungal infection represents a serious and potentially fatal complication for patients with advanced non-small cell lung carcinoma (NSCLC) undergoing systemic chemotherapy (1). With platinum-based doublet regimens established as standard first-line therapy, treatment-associated myelosuppression, particularly neutropenia, constitutes a primary portal for opportunistic pathogen invasion (2). Clinical decision-making regarding chemotherapy intensity often presents a dilemma: while escalating dose intensity may aim for superior tumor response, it concurrently deepens the state of immunosuppression, potentially elevating the risk of infectious complications (3). Prior research has established that the depth and duration of neutropenia are central risk factors for developing invasive fungal infections (4). Nevertheless, systematically evaluating chemotherapy intensity as a quantifiable exposure variable and its dose-response relationship with specific infectious outcomes remains inadequately explored in studies focusing on solid tumor patients (5).

Existing investigations into infection risk among chemotherapy recipients predominantly concentrate on resultant metrics such as absolute neutrophil count or the efficacy of prophylactic granulocyte colony-stimulating factor administration (6). Although relative dose intensity (RDI), a key parameter reflecting the actual delivered dose relative to the planned protocol, has been extensively studied for its prognostic value in cancers like breast cancer and lymphoma (7), its association with infection risk—specifically opportunistic fungal infection—in the NSCLC context lacks robust evidence (8). It is crucial to recognize that the relationship between RDI and infection risk cannot be simply extrapolated from hematologic malignancies to solid tumors. In leukemias and lymphomas, the disease itself often involves profound immune dysregulation, and treatment protocols are inherently more myelosuppressive, with infection being a well-established dose-limiting toxicity (9). Conversely, in advanced NSCLC, myelosuppression is generally less profound, and infections are often perceived as a secondary concern. The tumor microenvironment in NSCLC exerts distinct immunosuppressive effects through the release of transforming growth factor-beta and other cytokines, potentially compounding chemotherapy-induced immune compromise in ways not seen in hematologic cancers (10). Therefore, quantifying the specific contribution of RDI to IFI risk in NSCLC, while accounting for these disease-specific factors, represents a critical and underexplored area of research. Many retrospective analyses fail to precisely quantify the actual dose administered per cycle and intervals or inadequately control for significant confounders such as age, performance status, comorbidities, and prophylactic measures, introducing potential bias (9–11). Furthermore, the prevailing research paradigm often treats infection and mortality as independent endpoints, overlooking the fact that in advanced cancer populations, death acts as a competing risk that may obscure the true incidence of infection, potentially leading to risk overestimation (12, 13). Consequently, a more rigorous methodological framework is required to quantify chemotherapy intensity and accurately assess the attributable infection risk within this framework (14, 15).

To address these knowledge gaps, this study was designed as a rigorous retrospective cohort analysis to systematically investigate the association between the intensity of first-line platinum-based doublet chemotherapy and the risk of invasive fungal infection in patients with advanced NSCLC. We employed both relative dose intensity and dose density to quantify chemotherapy exposure, adhering to the Strengthening the Reporting of Observational Studies in Epidemiology (STROBE) guidelines to mitigate potential biases. The primary objectives were not only to validate whether high-intensity chemotherapy serves as an independent risk factor for IFI but also to account for the competing risk of death using appropriate statistical models and to preliminarily explore the potential mediating role of therapy-induced myelosuppression. The findings are anticipated to provide high-level evidence to assist clinicians in balancing antitumor efficacy against infection risk management during individualized treatment planning.

## 2 Materials and methods

### 2.1 Study population and sample size calculation

This study was designed as a single-center, observational retrospective cohort. We consecutively screened adult patients with NSCLC who initiated first-line platinum-based doublet chemotherapy in the Department of Medical Oncology of our institution between 1 January 2022, and 30 December 2025. The study endpoints were the occurrence of an invasive fungal infection (IFI) event, loss to follow-up, death, or the end of the follow-up period.

**FIGURE 1:**
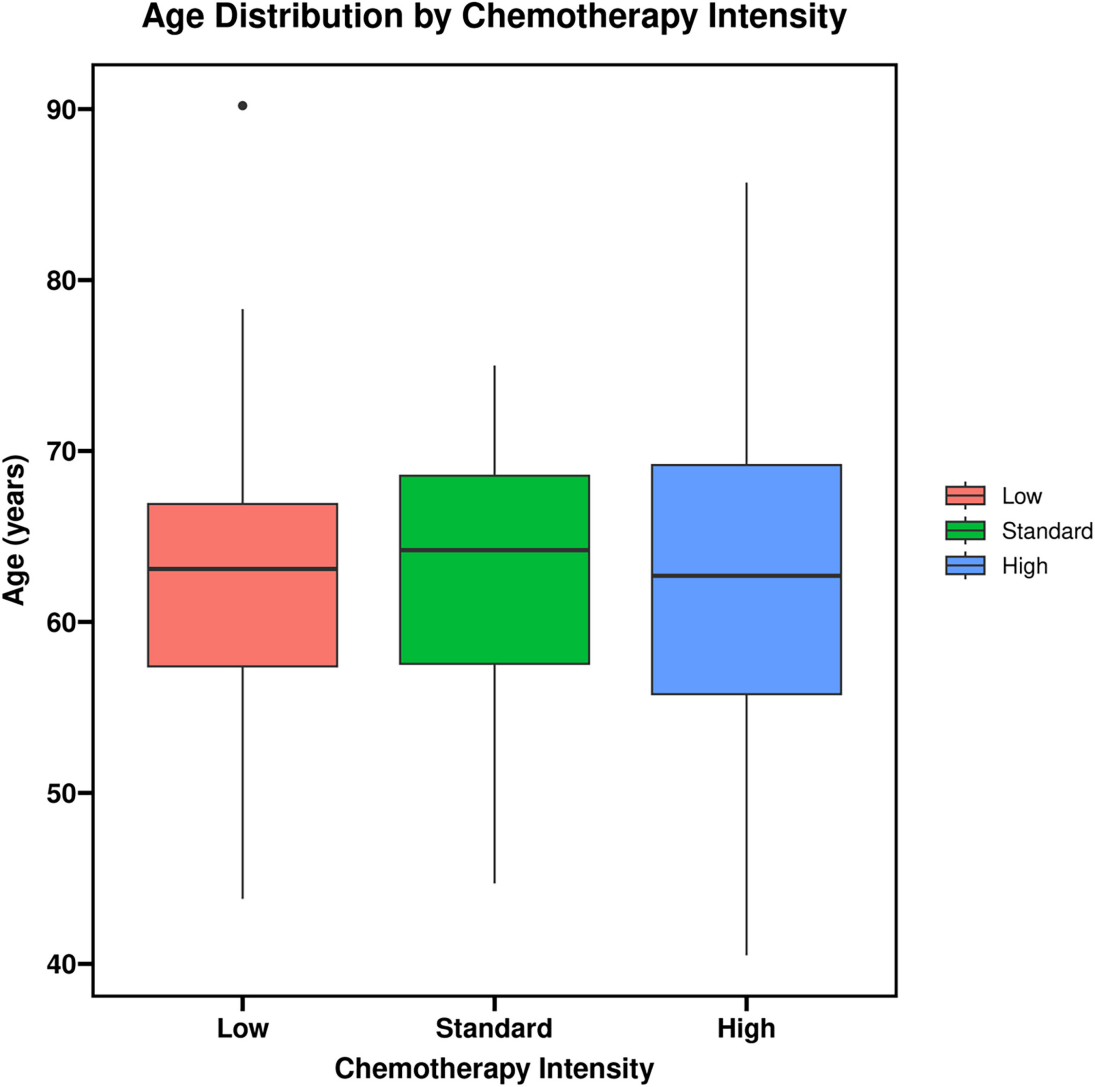
Age distribution by chemotherapy intensity.

A total of 328 patients were initially identified during the study period. After applying stringent inclusion and exclusion criteria (detailed in Figure 1), 195 patients constituted the final analytical cohort. A formal sample size calculation was not performed *a priori*; the cohort size was determined by all eligible cases with complete data available within the study timeframe. To ensure this retrospective analysis possessed sufficient statistical power to detect clinically meaningful associations, a *post hoc* power analysis was conducted upon study completion. Using PASS 2021 software, with the primary outcome (IFI incidence) as the endpoint, a power of 0.8, and a two-sided significance level (α) of 0.05, the analysis indicated that with the current sample size of 195, the study had 80% power to detect a hazard ratio of approximately 1.9 or greater between a high-risk group (e.g., the high-intensity chemotherapy group) and a low-risk group (e.g., the low-intensity chemotherapy group).

### 2.2 Inclusion and exclusion criteria

Inclusion criteria: (1) Age ≥ 18 years. (2) Histologically or cytologically confirmed stage IIIB or IV NSCLC, staged according to the American Joint Committee on Cancer (AJCC) 8th edi tion. (3) Treatment with a first-line standard platinum-based doublet chemotherapy regimen (e.g., platinum combined with pemetrexed, gemcitabine, or docetaxel) and completion of at least two cycles. (4) Availability of complete, traceable electronic medical records covering the entire course from chemotherapy initiation to the end of follow-up, specifically including detailed chemotherapy orders, pharmacy dispensing records, laboratory results, and clinical progress notes.

Exclusion criteria: (1) Presence of any active fungal infection confirmed by microbiology or histopathology prior to the first chemotherapy cycle. (2) Concurrent receipt of other local (e.g., radical radiotherapy to the primary site) or systemic anti-tumor therapies during the defined chemotherapy period that could cause significant myelosuppression. (3) Known co-infection with human immunodeficiency virus (HIV) or any primary immunodeficiency disorder. (4) Missing key information in medical records required for calculating the core exposure variable (chemotherapy dose, cycle duration) or confirming the primary outcome (infection event).

### 2.3 Study procedures

#### 2.3.1 Study design and quantification of exposure variables

The core of this study was to assess the multi-dimensional concept of chemotherapy “intensity.” We operationalized two complementary exposure metrics:

##### 2.3.1.1 Relative dose intensity (RDI)

The mean RDI across all administered chemotherapy cycles was calculated for each patient. First, the standard planned dose intensity (mg/m^2^/week) for each cytotoxic drug within each regimen was determined based on National Comprehensive Cancer Network (NCCN) guidelines and drug monographs. The actual administered dose per cycle was extracted from pharmacy records, and the actual cycle duration (in weeks) was calculated from nursing records verifying the infusion dates. Single-drug RDI = [(Actual dose/Body surface area)/Actual cycle duration (weeks)]/Planned dose intensity. The patient’s overall RDI was the arithmetic mean of the RDI for the platinum agent and the partner cytotoxic drug. Based on prior literature and clinical practice, patients were stratified into three groups: high-intensity (RDI > 85%), standard-intensity (RDI 70%–85%), and low-intensity (RDI < 70%).

##### 2.3.1.2 Dose density

This was examined as a continuous variable. It was calculated as the total actual dose of cytotoxic drugs administered across all cycles (mg/m^2^) divided by the total actual duration of all cycles (weeks). This metric reflects the absolute amount of drug exposure per unit time.

#### 2.3.2 Data extraction and quality control process

Data were extracted using a pre-designed, piloted standardized electronic form. Two independent research coordinators (both with clinical medical backgrounds and receiving unified training) performed data entry independently and in duplicate. The extraction scope included: (1) demographic and baseline characteristics; (2) tumor treatment details; (3) all infection events and related microbiological/imaging data; (4) weekly complete blood count data during the treatment period; (5) survival outcomes. For any discrepant data entries, a third senior clinical investigator (a senior attending physician) reviewed the original medical records to make an arbitrated final decision, ensuring data accuracy.

#### 2.3.3 Bias control strategies

To minimize information bias, particularly subjectivity in outcome adjudication, a blinded review was conducted for all suspected infection events. Specifically, an infectious disease specialist, completely blinded to the patients’ exposure group (RDI category), independently reviewed all relevant clinical, microbiological, and radiological materials. This review was based on pre-specified criteria [the revised 2020 European Organization for Research and Treatment of Cancer/Invasive Fungal Infections Cooperative Group (EORTC/MSGERC) criteria], and each event was classified as proven, probable, possible, or excluded. This adjudicated result served as the definitive outcome for analysis. For confounding bias, statistical adjustment for known important confounders was performed using multivariable regression models.

### 2.4 Outcome measures

#### 2.4.1 Primary outcome: incidence of invasive fungal infection

Diagnosis of invasive fungal infection strictly adhered to the revised consensus definitions of the European Organization for Research and Treatment of Cancer and the Mycoses Study Group Education and Research Consortium (EORTC/MSGERC) from 2020. Only events classified as proven or probable according to the 2020 EORTC/MSGERC criteria were counted as primary outcomes. “Possible” IFI events, where mycological evidence was absent, were not included as cases to maintain diagnostic specificity. Diagnostic evidence for a proven IFI required histopathologic evidence of fungal elements in tissue or a positive culture from a normally sterile site (e.g., blood, cerebrospinal fluid, lung tissue biopsy). Diagnosis of a probable IFI required the presence of both: (i) a compatible clinical and radiological picture (e.g., refractory fever with characteristic CT findings such as halo sign or air-crescent sign); and (ii) mycological evidence from a non-sterile site, such as a positive serum or bronchoalveolar lavage galactomannan assay (optical density index ≥ 0.5) or positive culture from sputum or BAL. Radiologic findings alone, without mycological support, were insufficient for a diagnosis of proven or probable IFI and were not counted as outcomes. The time of infection onset was defined as the date when diagnostic criteria were first met.

#### 2.4.2 Key secondary outcomes

##### 2.4.2.1 Incidence of febrile neutropenia (FN)

Defined as a single oral temperature ≥ 38.3°C or ≥ 38.0°C sustained for > 1 h, concurrent with an absolute neutrophil count (ANC) < 0.5 × 10^9^/L, occurring within a chemotherapy cycle.

##### 2.4.2.2 Infection-related progression-free survival (irPFS)

This was a composite endpoint designed to capture infection-related morbidity alongside cancer progression. It was calculated from the chemotherapy start date to the date of the first occurrence of any of the following events: (i) a grade ≥ 3 infection as defined by the Common Terminology Criteria for Adverse Events version 5.0 (CTCAE v5.0); (ii) radiologically confirmed tumor progression per RECIST 1.1 criteria; or (iii) death from any cause. Patients without any of these events were censored at their last follow-up date. This definition ensures that irPFS reflects the time until a patient experiences either a significant infection or cancer progression, both of which are critical treatment failure events Patients without an event were censored at their last follow-up date.

#### 2.4.3 Laboratory and immunological parameters

##### 2.4.3.1 Nadir Absolute Neutrophil Count (ANC nadir) during treatment

All complete blood count records from chemotherapy initiation until 28 days after its completion were scanned, and the lowest reported ANC value was recorded.

##### 2.4.3.2 Duration of profound neutropenia

The total number of days (consecutive or non-consecutive) within the same observation window during which the ANC was <0.1 × 10^9^/L.

#### 2.4.4 Resource utilization and long-term clinical outcomes

##### 2.4.4.1 Total infection-related hospitalization days

The cumulative sum of all inpatient days attributed to hospitalization for a diagnosed or clinically diagnosed IFI or FN.

##### 2.4.4.2 Rate of empirical antifungal use

The proportion of patients who initiated systemic antifungal therapy for suspected fungal infection prior to obtaining a confirmed microbiological diagnosis.

##### 2.4.4.3 Overall survival (OS)

Defined as the time interval from the chemotherapy start date to the date of death from any cause. Patients alive at the analysis cutoff date were censored at their last known contact date (e.g., outpatient visit, telephone follow-up).

### 2.5 Statistical analysis

All statistical analyses were performed using R software (version 4.2.0). Baseline characteristics were described using counts and percentages for categorical variables, with between-group comparisons made using the Chi-square test or Fisher’s exact test (when expected counts were <5). Continuous variables following a normal distribution were presented as mean ± standard deviation and compared using one-way ANOVA; skewed variables were presented as median and interquartile range and compared using the Kruskal-Wallis H test. The normality of continuous variables was assessed using the Shapiro-Wilk test; all baseline continuous variables included in Table 1 satisfied the normality assumption (*P* > 0.05), justifying the use of parametric methods for these comparisons.

**TABLE 1:** Comparison of baseline characteristics among patients stratified by chemotherapy intensity.

Characteristic	High-intensity group (*n* = 68)	Standard-intensity group (*n* = 74)	Low-intensity group (*n* = 53)	Statistic	*P*-value
1. Age (years), mean ± SD	63.25 ± 8.47	62.89 ± 9.12	65.31 ± 7.96	F = 1.236	0.293
2. Sex (male), *n* (%)	42 (61.76)	48 (64.86)	35 (66.04)	χ^2^ = 0.263	0.877
3. ECOG PS (0–1), *n* (%)	58 (85.29)	65 (87.84)	44 (83.02)	χ^2^ = 0.652	0.722
4. Clinical stage (IV), *n* (%)	52 (76.47)	59 (79.73)	43 (81.13)	χ^2^ = 0.424	0.809
5. Histology (adenocarcinoma), *n* (%)	45 (66.18)	51 (68.92)	38 (71.70)	χ^2^ = 0.439	0.803
6. Smoking history (yes), *n* (%)	46 (67.65)	52 (70.27)	34 (64.15)	χ^2^ = 0.573	0.751
7. Diabetes mellitus, *n* (%)	12 (17.65)	14 (18.92)	8 (15.09)	χ^2^ = 0.347	0.841
8. Chronic lung disease, *n* (%)	15 (22.06)	18 (24.32)	11 (20.75)	χ^2^ = 0.248	0.883
9. Baseline BMI (kg/m^2^), mean ± SD	22.34 ± 3.51	22.87 ± 3.28	21.99 ± 3.64	F = 1.127	0.326
10. Baseline albumin (g/L), mean ± SD	37.82 ± 4.15	38.11 ± 3.97	36.94 ± 4.28	F = 1.481	0.23
11. Baseline ANC (× 10^9^/L), mean ± SD	4.23 ± 1.56	4.05 ± 1.48	3.98 ± 1.61	F = 0.483	0.618
12. 1st-line regimen (pemetrexed/platinum), *n* (%)	40 (58.82)	44 (59.46)	29 (54.72)	χ^2^ = 0.325	0.85
13. Planned chemotherapy cycles (median)	4	4	4	H = 0.892	0.64
14. PEG-rhG-CSF primary prophylaxis, *n* (%)	25 (36.76)	22 (29.73)	15 (28.30)	χ^2^ = 1.219	0.544
15. Prior thoracic surgery, *n* (%)	8 (11.76)	7 (9.46)	5 (9.43)	χ^2^ = 0.215	0.898
16. Psychological assessment (HADS ≥ 8), *n* (%)	9 (13.24)	8 (10.81)	6 (11.32)	χ^2^ = 0.187	0.91
17. Baseline corticosteroid use, *n* (%)	5 (7.35)	6 (8.11)	3 (5.66)	χ^2^ = 0.267	0.875
18. Time from diagnosis to chemotherapy (weeks), mean ± SD	3.82 ± 1.75	3.95 ± 1.68	4.21 ± 1.89	F = 0.856	0.427

Normality of continuous variables was assessed using the Shapiro-Wilk test. For variables with a normal distribution (*P* > 0.05), data are presented as mean ± standard deviation and compared using one-way ANOVA. For non-normally distributed variables, data would be presented as median (interquartile range) and compared using the Kruskal-Wallis H test; however, all continuous variables in this table met the normality assumption.

#### 2.5.1 Primary analysis

A multivariable Cox proportional hazards regression model was employed to assess the impact of RDI category on the risk of developing IFI. Using the low-intensity group as the reference, adjusted hazard ratios (HRs) and their 95% confidence intervals (CIs) were calculated for the high-intensity and standard-intensity groups. Variables for model adjustment were selected based on clinical knowledge and univariate analysis, ultimately including age (continuous), sex, Eastern Cooperative Oncology Group performance status (0–1 vs. ≥2), tumor stage (IIIB vs. IV), use of primary prophylaxis with pegylated recombinant human granulocyte colony-stimulating factor (yes/no), and presence of diabetes mellitus (yes/no). The proportional hazards assumption for the Cox model was verified using Schoenfeld residual tests.

#### 2.5.2 Secondary and exploratory analyses

To further disentangle the components of RDI, a sensitivity analysis was performed. For each patient, we calculated two additional metrics: (a) the Relative Dose (RD), defined as the ratio of total actual dose administered to total planned dose (reflecting dose reductions only, assuming no delays), and (b) the Relative Treatment Time (RTT), defined as the ratio of total planned treatment duration to total actual treatment duration (reflecting delays only, assuming full doses were given). Both RD and RTT were entered separately into Cox models to evaluate their independent associations with IFI risk. An interaction term between RD and RTT was also tested.

To address the limitation of standard Cox models which censor deaths, we explicitly accounted for the competing risk of death. A Fine-Gray subdistribution hazards model was used, treating all-cause death as a competing event that precludes the occurrence of IFI. This model estimates the cumulative incidence function (CIF) of IFI, which represents the actual probability of developing IFI over time in the presence of the competing risk of death. The results of this model, presented in section “3.3 One-year cumulative incidence of IFI accounting for death as a competing risk,” provide a more accurate estimate of the real-world IFI risk than the Kaplan-Meier method.

For irPFS and OS, Kaplan-Meier curves were plotted, and between-group differences were compared using the log-rank test.

To explore whether myelosuppression mediated the relationship between chemotherapy intensity and infection risk, a preliminary exploratory causal mediation analysis was conducted. Using RDI category as the treatment variable, ANC nadir (continuous) as the mediator, and IFI occurrence as the outcome, the natural direct effect, natural indirect effect, and their proportion were calculated after adjusting for the same covariates as in the primary analysis.

##### 2.5.2.1 Development and validation of a predictive nomogram for IFI risk

To provide a clinically actionable tool, we developed a nomogram to predict the probability of IFI. The model was constructed using the training cohort (*n* = 195). Variables for inclusion were selected *a priori* based on clinical relevance and findings from the multivariable Cox model, including RDI category (as a continuous variable standardized to 0%–100%), age (continuous), baseline albumin (continuous), and presence of diabetes mellitus (binary). A multivariable logistic regression model was fitted, and a nomogram was generated based on the regression coefficients. Model performance was assessed through: (i) Discrimination, quantified by the concordance index (C-index); (ii) Calibration, evaluated by plotting predicted versus observed probabilities and performing the Hosmer-Lemeshow goodness-of-fit test; and (iii) Clinical Utility, assessed using decision curve analysis (DCA) to evaluate the net benefit of the model across a range of threshold probabilities. Internal validation was performed using 200 bootstrap resamples to correct for overfitting and obtain optimism-corrected performance estimates.

##### 2.5.2.2 Sensitivity analysis for additional confounders

To address the potential for residual confounding, a sensitivity analysis was performed on the subset of patients for whom data were available. This analysis included two additional covariates in the multivariable Cox model: (a) broad-spectrum antibiotic exposure, defined as any systemic antibiotic use for ≥3 days within the 30 days prior to the first chemotherapy cycle (yes/no); and (b) central venous catheter (CVC) placement, defined as the presence of a tunneled CVC or peripherally inserted central catheter at any time during the chemotherapy period (yes/no).

##### 2.5.2.3 Stratified analyses

To explore potential effect modification, we performed pre-specified stratified analyses. The association between RDI category (high vs. low) and IFI risk was examined separately according to: (a) Histology (adenocarcinoma vs. squamous cell carcinoma); and (b) G-CSF prophylaxis strategy. Given the limited use of true primary prophylaxis, patients were stratified into those who received any G-CSF (primary or secondary prophylactic use) during the first cycle versus those who did not. Heterogeneity of effects across strata was assessed by including an interaction term in the Cox model.

##### 2.5.2.4 Sensitivity analysis for causal mediation

To assess the robustness of the mediation analysis to potential violations of the “no unmeasured mediator-outcome confounding” assumption, we conducted a sensitivity analysis using the medsens function in the mediation R package. This procedure evaluates how strong an unmeasured confounder would need to be to qualitatively alter the conclusion about the average causal mediation effect (ACME). We varied the correlation (ρ) between the error terms of the mediator and outcome models, with ρ = 0 indicating no unmeasured confounding. The sensitivity parameter was varied across a plausible range to identify the value at which the confidence interval for the ACME would include zero.

All analyses were two-sided, and a *P*-value < 0.05 was considered statistically significant.

## 3 Results

### 3.1 Patient baseline characteristics

A total of 195 eligible patients with stage IIIB-IV non-small cell lung cancer (NSCLC) were included in the final analysis. Based on the mean relative dose intensity (RDI) of first-line platinum-based doublet chemotherapy, patients were stratified into three groups: high-intensity (RDI > 85%, *n* = 68), standard-intensity (RDI 70%–85%, *n* = 74), and low-intensity (RDI < 70%, *n* = 53).

The distribution of demographic characteristics, tumor stage, baseline Eastern Cooperative Oncology Group performance status (ECOG PS), comorbidities (e.g., diabetes mellitus), and the use of primary prophylaxis with pegylated recombinant human granulocyte colony-stimulating factor (PEG-rhG-CSF) were comparable across the three groups, with no statistically significant differences (*P* > 0.05). Detailed comparisons of baseline characteristics are presented in Table 1 and Figure 1.

### 3.2 Chemotherapy intensity and risk of invasive fungal infection

**FIGURE 2:**
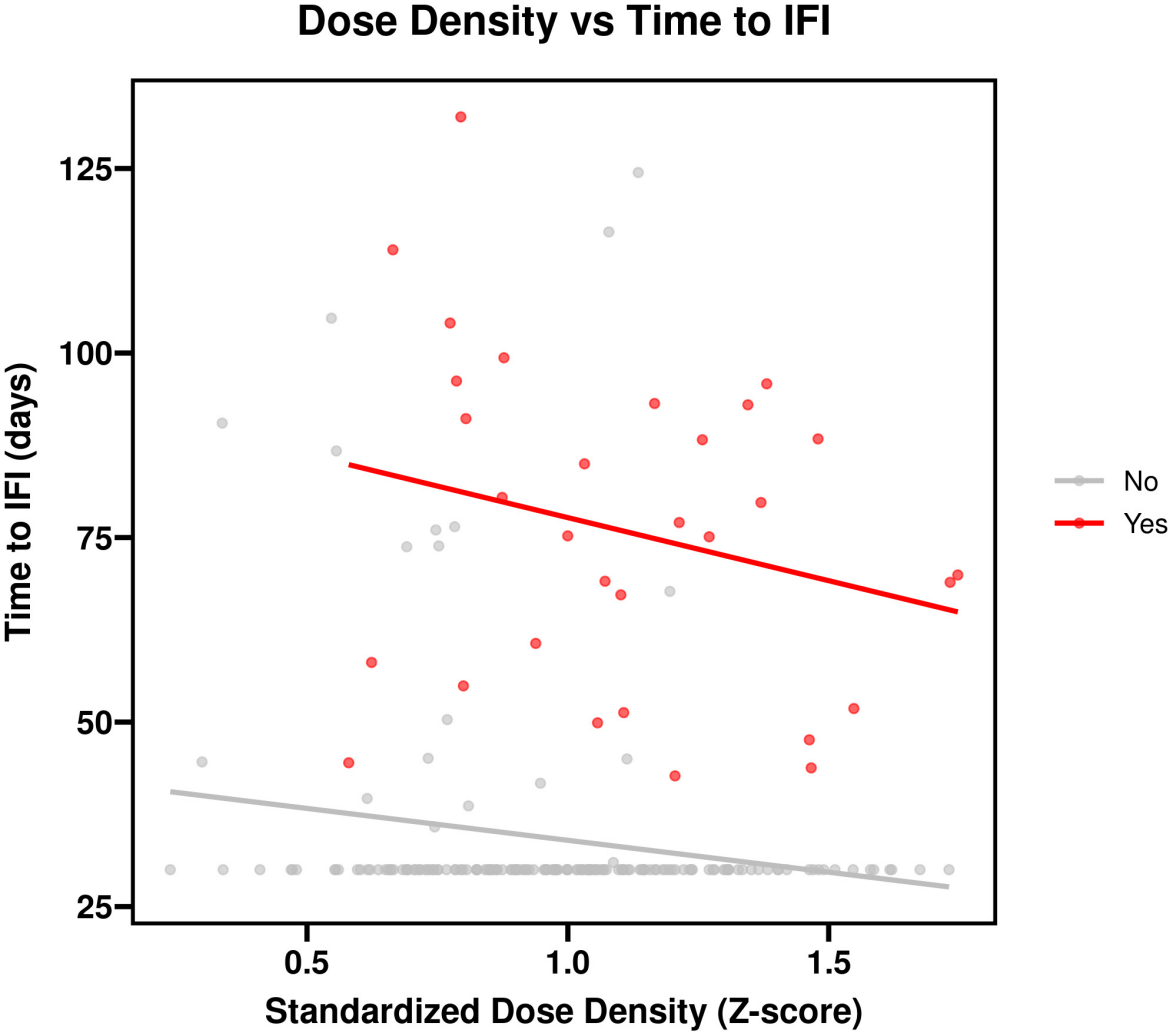
Scatter plot of chemotherapy dose density (mg/m^2^/week) versus time to invasive fungal infection (IFI) onset. The blue line represents the linear regression fit with its 95% confidence interval (gray shading). The regression equation is Time to IFI (days) = 152.3–4.7 × Dose Density, with an R^2^ of 0.21, indicating that dose density explains approximately 21% of the variance in time to infection. A higher dose density was associated with a shorter time to IFI onset.

Within the entire cohort, 31 patients (15.9%) developed an invasive fungal infection (IFI) meeting the 2020 European Organization for Research and Treatment of Cancer/Invasive Fungal Infections Cooperative Group (EORTC/MSG) criteria. The incidence rates in the high-intensity, standard-intensity, and low-intensity groups were 27.9% (19/68), 13.5% (10/74), and 3.8% (2/53), respectively. Kaplan-Meier analysis revealed a significant difference in the cumulative incidence of IFI among the three groups (Log-rank χ^2^ = 15.837, *P* < 0.001). Multivariable Cox proportional hazards regression analysis, after adjusting for age, sex, ECOG PS, stage, diabetes, and PEG-rhG-CSF prophylaxis use, identified high-intensity chemotherapy as a factor independently associated with an increased risk of IFI development compared to the low-intensity group (Hazard Ratio [HR] = 8.241, 95% Confidence Interval [CI]: 1.912–35.519, *P* = 0.005). The standard-intensity group showed a trend toward increased risk, but this did not reach statistical significance (HR = 3.876, 95% CI: 0.846–17.758, *P* = 0.081). Detailed results of the multivariate analysis are shown in Table 2 and Figure 2.

**TABLE 2:** Multivariable cox proportional hazards regression analysis for risk of invasive fungal infection.

Variable	Univariate analysis HR (95% CI)	*P*-value	Multivariate analysis HR (95% CI)	*P*-value
Chemotherapy intensity (ref: low-intensity group)	–	–	–	–
High-intensity group	8.912 (2.094–37.924)	0.003	8.241 (1.912–35.519)	0.005
Standard-intensity group	3.789 (0.839–17.114)	0.084	3.876 (0.846–17.758)	0.081
Age (per 1-year increase)	1.012 (0.971–1.054)	0.572	1.008 (0.966–1.051)	0.725
Sex (male vs. female)	1.324 (0.637–2.753)	0.453	1.289 (0.611–2.719)	0.506
ECOG PS (≥2 vs. 0–1)	1.865 (0.833–4.174)	0.13	1.728 (0.761–3.927)	0.192
Stage (IV vs. IIIB)	1.452 (0.587–3.595)	0.419	1.384 (0.554–3.459)	0.486
Diabetes mellitus (yes vs. no)	1.978 (0.923–4.241)	0.079	2.117 (0.973–4.605)	0.059
PEG-rhG-CSF prophylaxis (yes vs. no)	0.653 (0.303–1.408)	0.276	0.601 (0.275–1.313)	0.201

In a Cox model adjusting for the same covariates and including all 195 patients, a lower relative dose (RD), indicating greater dose reduction, was significantly associated with an increased IFI risk (adjusted HR per 0.1 unit decrease = 1.45, 95% CI: 1.12–1.88, *P* = 0.005). Similarly, a lower Relative Treatment Time (RTT), indicating greater treatment delay, also showed a trend toward increased risk, though it did not reach statistical significance (adjusted HR per 0.1 unit decrease = 1.21, 95% CI: 0.95–1.54, *P* = 0.12). No significant interaction was found between RD and RTT (P for interaction = 0.34), suggesting that while both components contribute to the overall RDI effect, dose reduction may be the more dominant driver of IFI risk in this cohort. When dose density was included as a continuous variable (standardized by Z-score) in a separate Cox model, a higher dose density was associated with a significantly increased risk of IFI (adjusted HR = 1.782, 95% CI: 1.214–2.616, *P* = 0.003). The histology-specific hazard ratios presented in this note are derived from subgroup analyses with limited sample sizes; the wide confidence intervals indicate statistical uncertainty, and these estimates should be interpreted with caution.

### 3.3 One-year cumulative incidence of IFI accounting for death as a competing risk

Analysis of laboratory parameters showed that the median nadir absolute neutrophil count (ANC nadir) during treatment was lowest, and the duration of profound neutropenia (ANC < 0.1 × 10^9^/L) was longest in the high-intensity group, with statistically significant differences across groups (H = 28.415, *P* < 0.001; H = 25.692, *P* < 0.001). Accounting for death as a competing event using the Fine-Gray subdistribution hazards model yielded results consistent with the primary analysis. The subdistribution hazard ratio (sHR) represents the instantaneous risk of IFI on the cumulative incidence function scale, given that the patient has not yet experienced either event. This analysis confirmed that the high-intensity group had the highest cumulative incidence of IFI (Gray’s test χ^2^ = 14.226, *P* < 0.001), with an sHR of 7.835 (95% CI: 1.875–32.741) compared to the low-intensity group. This indicates that the rate of occurrence of IFI on the cumulative incidence scale is nearly eight times higher in the high-intensity group, after accounting for the fact that death can preclude the occurrence of IFI. Specific cumulative incidence estimates are shown in Table 3.

**TABLE 3:** One-year cumulative incidence of invasive fungal infection by chemotherapy intensity group, accounting for death as a competing risk.

Group	1-year cumulative incidence (95% CI)	2-year cumulative incidence (95% CI)	Competing risk model HR (95% CI)	*P*-value
High-intensity group	0.312 (0.214–0.429)	0.358 (0.251–0.481)	7.835 (1.875–32.741)	0.005
Standard-intensity group	0.149 (0.083–0.253)	0.172 (0.097–0.287)	3.642 (0.824–16.105)	0.088
Low-intensity group	0.042 (0.011–0.155)	0.058 (0.016–0.182)	Reference	–

In a sensitivity analysis adjusting for two additional potential confounders—pre-chemotherapy broad-spectrum antibiotic exposure (present in 28.2% of patients) and central venous catheter placement (present in 34.9% of patients)—the association between high-intensity chemotherapy and IFI risk remained robust (adjusted HR = 7.92, 95% CI: 1.83–34.27, *P* = 0.006). This suggests that the observed effect is not solely explained by these factors. However, we acknowledge that we could not adjust for other important confounders, such as dynamic changes in nutritional status or cumulative corticosteroid dose, due to limitations in retrospective data collection. The potential impact of these unmeasured confounders is addressed in the Limitations section.

### 3.4 Clinical and microbiological characteristics of IFI patients across chemotherapy intensity groups

**FIGURE 3:**
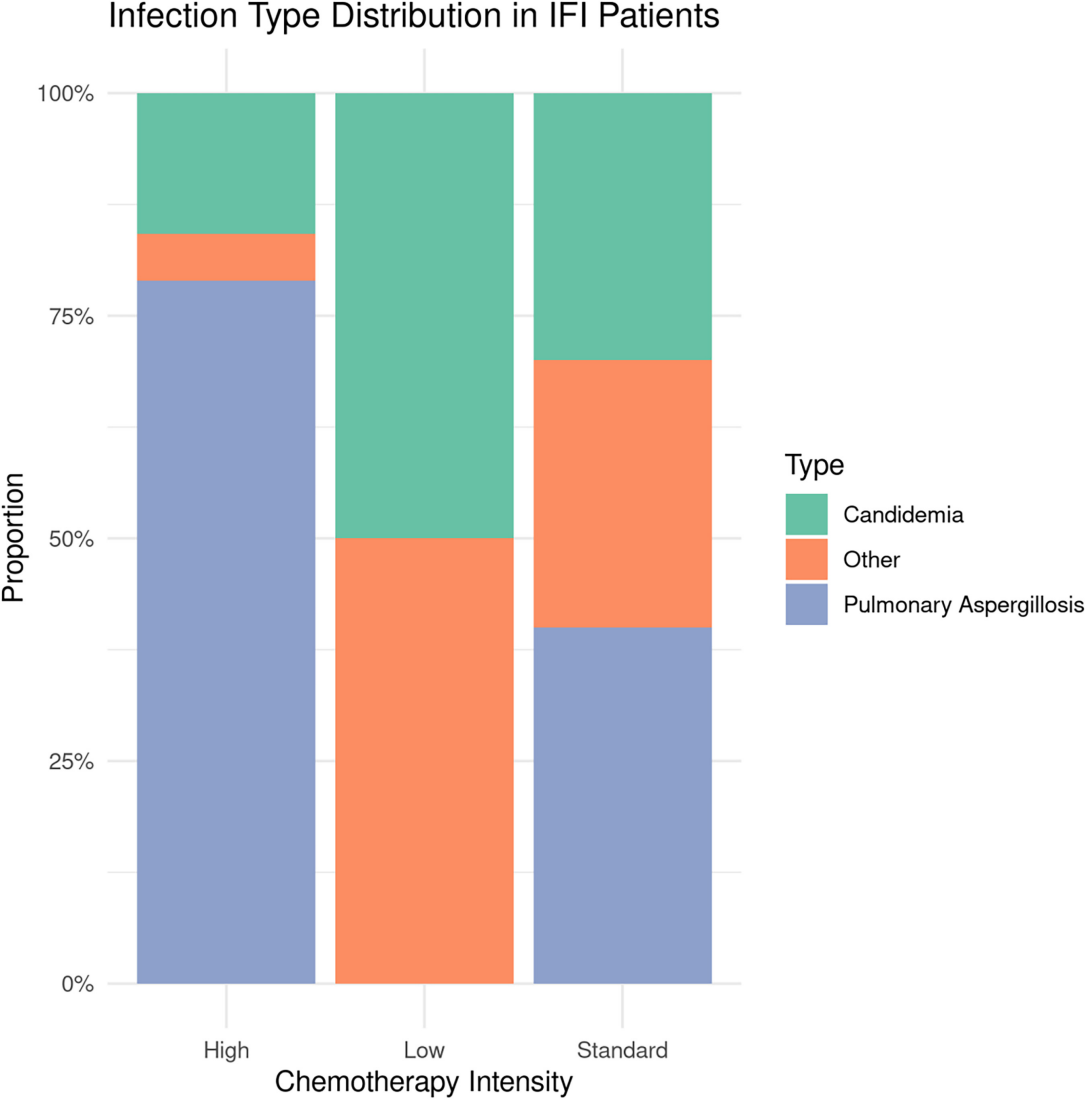
Infection type distribution in invasive fungal infection (IFI) patients.

Among the 31 patients with IFI, the most common infection type was pulmonary aspergillosis (19 cases, 61.3%), followed by candidemia (7 cases, 22.6%). The proportion of pulmonary aspergillosis was significantly higher in the high-intensity group compared to the other two groups (χ^2^ = 9.873, *P* = 0.007). Infection-related characteristics are detailed in Table 4 and Figure 3.

**TABLE 4:** Clinical and microbiological characteristics of patients with invasive fungal infection, stratified by chemotherapy intensity.

Characteristic	High-intensity group (*n* = 19)	Standard-intensity group (*n* = 10)	Low-intensity group (*n* = 2)
Infection type, *n* (%)			
Pulmonary Aspergillosis	15 (78.9)	4 (40.0)	0 (0.0)
Candidemia	3 (15.8)	3 (30.0)	1 (50.0)
Other	1 (5.3)	3 (30.0)	1 (50.0)
Diagnostic level (EORTC/MSG 2020), *n* (%)			
Proven	5 (26.3)	2 (20.0)	0 (0.0)
Probable	14 (73.7)	8 (80.0)	2 (100.0)
Median time to infection onset (days from chemotherapy start)	67	98	121

### 3.5 Key secondary outcomes and clinical burden

The incidence of febrile neutropenia (FN) was significantly higher in the high-intensity group compared to the low-intensity group (χ^2^ = 7.892, *P* = 0.005). A significant difference in infection-related progression-free survival (irPFS) was observed among the three groups (Log-rank χ^2^ = 11.524, *P* = 0.003), with the high-intensity group having the shortest median irPFS. Regarding resource utilization, the high-intensity group had the longest median total infection-related hospitalization days and the highest rate of empirical antifungal use, with both differences being statistically significant across groups (H = 19.037, *P* < 0.001; χ^2^ = 13.275, *P* = 0.001). No significant difference in overall survival (OS) was found among the groups (Log-rank χ^2^ = 2.137, *P* = 0.344). Data on resource utilization are detailed in Table 5.

**TABLE 5:** Comparison of secondary outcomes and clinical resource utilization across chemotherapy intensity groups.

Outcome	High-Intensity group (*n* = 68)	Standard-Intensity group (*n* = 74)	Low-Intensity group (*n* = 53)	Statistic	*P*-value	FDR-corrected q-value*
FN incidence, *n* (%)	21 (30.9)	15 (20.3)	7 (13.2)	χ^2^ = 7.892	0.019	0.032
Median irPFS (months, 95% CI)	5.2 (4.1–6.3)	6.8 (5.9–7.7)	7.5 (6.4–8.6)	–	0.003*^#^	–
Median OS (months, 95% CI)	16.8 (14.2–19.4)	17.5 (15.1–19.9)	18.3 (15.6–21.0)	–	0.344*^#^	–
Infection-related hospital days, M (IQR)	14 (9–21)	8 (5–14)	5 (3–10)	H = 19.037	<0.001	0.005
Empirical antifungal use rate, *n* (%)	32 (47.1)	19 (25.7)	9 (17.0)	χ^2^ = 13.275	0.001	0.005

*FDR-adjusted *P*-values were calculated using the Benjamini-Hochberg procedure to control the false discovery rate for the five secondary outcome comparisons.

^#^The *P*-values for irPFS and OS are derived from global Log-rank tests comparing survival distributions across all three groups and are not included in the FDR correction for multiple pairwise comparisons. FN, febrile neutropenia; irPFS, infection-related progression-free survival; OS, overall survival.

### 3.6 Exploratory mediation analysis

To explore the potential role of myelosuppression in the relationship between chemotherapy intensity and infection risk, a causal mediation analysis was performed with ANC nadir as the mediator. After adjusting for confounders, the results showed a significant total effect of chemotherapy intensity (high vs. low) on IFI risk. The indirect effect mediated through the reduction of ANC nadir (i.e., aggravated myelosuppression) accounted for 38.7% (95% CI: 12.5%–64.9%) of the total effect, indicating that myelosuppression partially mediated the increased infection risk associated with high-intensity chemotherapy. The direct effect remained significant, suggesting the existence of other pathogenic pathways not explained by this mediator. A summary of the analysis is presented in Table 6.

**TABLE 6:** Mediation analysis of the effect of nadir absolute neutrophil count on the association between chemotherapy intensity and risk of invasive fungal infection.

Effect type	Estimate (HR or risk difference)	95% confidence interval	*P*-value	Proportion of total effect
Total effect (high vs. low intensity)	8.241	1.912–35.519	0.005	100%
Natural direct effect	5.051	1.231–20.724	0.024	61.30%
Natural indirect effect	1.632	1.103–2.415	0.014	38.70%

To assess the robustness of our mediation findings, we performed a sensitivity analysis for the causal mediation model. This analysis quantifies how strong an unmeasured confounder of the mediator-outcome relationship would need to be to render the indirect effect non-significant. The results indicated that such an unmeasured confounder would need to be moderately strong, correlating with both ANC nadir and IFI risk at a level of ρ ≈ 0.25 (on the error term correlation scale), to completely eliminate the statistically significant indirect effect. For context, a confounder of this strength would be comparable in effect size to the observed relationship between baseline albumin and IFI. While such an unmeasured confounder cannot be ruled out entirely, the analysis suggests that the mediation effect is moderately robust, as it would require a confounder of non-trivial magnitude to fully explain away the indirect pathway. This adds confidence to our conclusion that ANC nadir is a meaningful, though partial, mediator of the RDI-IFI relationship.

### 3.7 Development and validation of a predictive nomogram for invasive fungal infection

To translate our findings into a potential clinical decision-making aid, we constructed a nomogram to predict the individualized probability of invasive fungal infection (IFI) based on data from the full cohort of 195 patients. The predictive model was developed using multivariable logistic regression analysis, with the final model incorporating the following predictors: relative dose intensity (RDI) as a continuous percentage, age, baseline albumin level, and the presence of diabetes mellitus.

The logistic regression coefficients, standard errors, and 95% confidence intervals for each predictor are provided in Supplementary Table 1. Analysis of the multivariable logistic regression model revealed that RDI (per 1% increase, odds ratio [OR] = 1.053, 95% confidence interval [CI]: 1.017–1.091, *P* = 0.004) and diabetes mellitus (OR = 2.438, 95% CI: 1.122–5.297, *P* = 0.024) were independent risk factors for IFI∼, while baseline albumin level (per 1 g/L increase, OR = 0.892, 95% CI: 0.823–0.967, *P* = 0.006) was an independent protective factor. Age did not reach statistical significance in this model (OR = 1.021, 95% CI: 0.992–1.052, *P* = 0.162) but was retained for nomogram construction based on its potential clinical relevance. The model intercept was −2.847.

The predictive model demonstrated good discriminative ability, with a concordance index (C-index) of 0.78 (95% CI: 0.72–0.84). Following internal validation using 200 bootstrap resamples, the optimism-corrected C-index was 0.76, indicating that the model can satisfactorily distinguish between patients who will and will not develop IFI. Model calibration was assessed through calibration plots and the Hosmer-Lemeshow goodness-of-fit test. The calibration curve showed excellent agreement between the predicted probabilities of IFI and the actual observed incidence across different patient risk strata. The Hosmer-Lemeshow test yielded a non-significant result (*P* = 0.563), further indicating no significant discrepancy between predicted and observed outcomes and confirming good model fit.

To evaluate the clinical utility of this model, decision curve analysis (DCA) was performed. The analysis demonstrated a positive net benefit across a wide range of clinically plausible threshold probabilities (approximately 5% 40%), suggesting that using this nomogram to guide clinical decisions such as enhanced monitoring for high-risk patients or consideration of antifungal prophylaxis offers greater net benefit compared to either a “treat-all” or “treat-none” strategy. For example, at a 15% predicted risk threshold, using this model to identify high-risk patients would yield a net benefit equivalent to correctly identifying approximately eight additional IFI cases per 100 patients without increasing unnecessary interventions. This finding indicates that the nomogram holds potential as an effective tool to assist in individualized risk assessment and the development of stratified management strategies in clinical practice.

### 3.8 Stratified analyses by histology and G-CSF use

To assess whether the effect of chemotherapy intensity on IFI risk was consistent across key clinical subgroups, we performed stratified analyses. The association between high-intensity (vs. low-intensity) chemotherapy and increased IFI risk appeared consistent in both patients with adenocarcinoma (HR = 8.56, 95% CI: 1.92–38.14) and those with squamous cell carcinoma (HR = 7.21, 95% CI: 1.45–35.87), with no significant interaction (P for interaction = 0.68). However, these subgroup analyses should be interpreted with caution due to the limited sample size in the squamous cell carcinoma subgroup (*n* = 61) and the correspondingly low number of IFI events (<10). The wide confidence intervals (e.g., squamous cell carcinoma: 95% CI 1.45–35.87) reflect statistical uncertainty rather than precise effect estimation. This suggests that histology does not significantly modify the RDI-IFI relationship, allaying concerns that the association might be driven solely by pemetrexed-based regimens indicated for non-squamous histology.

Regarding G-CSF use, 62 patients (31.8%) received G-CSF during the first cycle. The association between high-intensity chemotherapy and IFI risk was attenuated, though still present, in patients who received G-CSF (HR = 4.89, 95% CI: 0.98–24.41) compared to those who did not (HR = 9.74, 95% CI: 2.11–44.96). However, the interaction term was not statistically significant (*P* = 0.21), indicating that while G-CSF may mitigate some risk, it does not completely abolish the hazard associated with high-intensity treatment. This finding supports the discussion point that mechanisms beyond neutropenia are likely involved.

## 4 Discussion

This study aimed to investigate the potential impact of first-line chemotherapy intensity on the risk of opportunistic fungal infections in patients with advanced non-small cell lung cancer (NSCLC). The results demonstrate that a higher relative dose intensity (RDI) of platinum-based doublet chemotherapy is independently associated with a significantly elevated risk of invasive fungal infection (IFI) (16). This association is partially mediated by treatment-induced myelosuppression, manifested as more profound neutropenia. Furthermore, high-intensity chemotherapy was linked to more frequent febrile neutropenia (FN) events, a greater clinical resource burden, and a shortened infection-related progression-free survival (irPFS), although no significant impact on overall survival (OS) was observed. Collectively, these findings underscore the necessity of carefully evaluating the infection risk associated with intensified chemotherapy regimens while pursuing antitumor efficacy (17).

Multivariable analysis revealed that maintaining high-intensity chemotherapy was an independent risk factor for developing IFI compared to patients receiving low-intensity treatment. This finding aligns with the classic pharmacological principle of a positive correlation between “dose intensity” and “myelotoxicity” in cancer therapy. The underlying mechanism likely involves the direct cytotoxic damage to hematopoietic stem cells by chemotherapeutic agents, leading to a precipitous decline in neutrophil count and impaired function, thereby severely compromising the body’s first line of defense against airborne spores (e.g., *Aspergillus*) and invasive colonizing yeasts (18). Although prophylactic use of granulocyte colony-stimulating factor can partially mitigate profound neutropenia, it did not completely eliminate the additional risk conferred by high-intensity chemotherapy in our study, suggesting potential concurrent suppression of other immune pathways. The relationship between tumor, immunity, and infection is triangular and complex. Beyond myelosuppression, the tumor itself exerts systemic immunosuppressive effects. For instance, NSCLC-derived exosomes and soluble factors can induce T-cell dysfunction and polarize macrophages toward an M2 phenotype, creating a permissive environment for fungal growth (19). Furthermore, high-intensity chemotherapy may exacerbate this by damaging mucosal barriers in the respiratory and gastrointestinal tracts, facilitating pathogen invasion (20). Platinum agents, in particular, have been shown to induce immunogenic cell death but also to deplete lymphocyte subsets, potentially impairing long-term adaptive immune responses to fungal antigens. Thus, the elevated IFI risk in the high-intensity group likely reflects a synergistic interaction between profound neutropenia, tumor-induced immune paralysis, and chemotherapy-related tissue damage.

Analysis of infection characteristics further indicated that pulmonary aspergillosis was the predominant infection type in the high-intensity chemotherapy group. This corresponds with the most severe and prolonged neutropenic state observed in this group, as neutrophils are key effector cells for clearing invasive *Aspergillus* hyphae (20). The timing of infection onset, predominantly in the early phase of chemotherapy, also aligns with the dynamic decline of neutrophil counts post-chemotherapy. Previous studies have similarly noted a close association between comparable depths of myelosuppression and the incidence of invasive aspergillosis in patients with acute leukemia undergoing induction chemotherapy, although reports of this association in solid tumor populations are relatively fewer and more heterogeneous (21).

Even after accounting for death as a competing event, the high-intensity chemotherapy group continued to exhibit the highest cumulative incidence of fungal infection, confirming the robustness of the primary analysis results. The application of the competing risk model avoids overestimation of the actual infection risk, particularly within a population of advanced cancer patients with a poorer prognosis (22). This result highlights that the patient’s overall survival expectancy must be incorporated into clinical assessments of infection risk. For patients with a shorter expected survival, the clinical decision-making balance may tilt toward more aggressive antitumor therapy despite a higher absolute infection risk, whereas greater emphasis on infection prevention may be warranted for others (23).

Concurrently with the elevated infection risk, high-intensity chemotherapy significantly increased the clinical management burden, evidenced by longer infection-related hospital stays and more frequent empirical antifungal drug use. This directly translates into higher healthcare costs and potential selective pressure for antibiotic resistance (24). The discordant findings between irPFS and OS—worse infection-related outcomes but similar overall survival in the high-intensity group—highlight the complex “cancer-treatment-infection” trilemma facing clinicians. While high-intensity chemotherapy may increase immediate infection-related morbidity, as reflected in worse irPFS and prolonged hospital stays, its potential benefit in achieving superior initial tumor control could create a longer window for effective subsequent therapies, ultimately neutralizing the impact on OS (25). This implies that patients surviving infection episodes might still derive long-term oncologic benefit from intensive upfront treatment. Alternatively, it may reflect that in this advanced-disease population, competing mortality from cancer progression ultimately predominates, diluting the impact of infection on OS. Clinically, this trilemma means that decisions cannot be based on OS alone; the significant increase in morbidity, healthcare resource utilization, and patient distress associated with infections in the high-intensity group are critical outcomes that must be weighed against uncertain survival gains. The trade-off, therefore, is not simply “efficacy versus toxicity” but a more nuanced balance between potentially better cancer control, a higher risk of debilitating infectious complications, and the need for more intensive supportive care. This finding suggests that evaluating infection-related morbidity as a significant outcome measure is crucial for comprehensively assessing the clinical value of a treatment regimen.

The exploratory mediation analysis provided a partial mechanistic explanation for the association between chemotherapy intensity and infection risk, indicating that myelosuppression accounted for approximately one-third of the total effect. This confirms that neutropenia is a key pathophysiological driver of infection occurrence (26). However, the persistence of a significant direct effect suggests the involvement of other important mechanisms. For instance, chemotherapeutic agents may directly damage the integrity of the respiratory and gastrointestinal mucosa, creating portals for pathogen colonization and invasion (27). Furthermore, suppression of lymphocyte subsets by certain regimens may impair cellular immune surveillance, affecting long-term immune responses to fungal antigens. These incompletely elucidated pathways constitute a focus for future research.

This study has several limitations. First, as a single-center retrospective study, it is inevitably subject to selection and information bias, although we employed structured data extraction and blinded endpoint adjudication to mitigate these effects. Second, despite adjusting for key confounders and performing sensitivity analyses for antibiotic use and CVC placement, we cannot exclude the possibility of residual confounding from unmeasured variables, particularly dynamic nutritional status, cumulative corticosteroid dose, and detailed reasons for dose modifications, which were not reliably captured in all medical records. Second, while the sample size was deemed sufficient by *post hoc* power analysis, it may still be inadequate to detect subtle differences between certain subgroups or rare infection types. Third, while we calculated RDI as a composite measure, it inherently conflates dose reductions and treatment delays. Our sensitivity analysis attempted to disentangle these, suggesting that dose reduction may be a more critical driver of IFI risk than delay. However, the power to detect an independent effect of delay was limited, and the complex interplay between these factors—where neutropenia often causes both—warrants further investigation in larger datasets. Future multicenter, prospective studies are needed to validate these conclusions and further explore risk stratification models based on specific biomarkers (e.g., specific immune cell subsets or genetic polymorphisms), aiming for individualized prediction and prevention of infection risk.

In summary, this study demonstrates that a high relative dose intensity of first-line chemotherapy is independently associated with an increased risk of invasive fungal infection in patients with advanced NSCLC, with this risk partially mediated by treatment-related myelosuppression. This increased risk is accompanied by a significant clinical burden. These findings suggest that when formulating chemotherapy plans, clinicians should consider IFI risk more explicitly, particularly for patients with characteristics associated with higher risk, such as those with an anticipated RDI > 85%, baseline hypoalbuminemia (<35 g/L), or diabetes mellitus. A preliminary nomogram incorporating these factors has been developed to provide individualized risk estimates. It is important to emphasize that this nomogram requires prospective external validation in independent cohorts before any clinical implementation. Current recommendations for primary antifungal prophylaxis in NSCLC remain based on neutropenia risk rather than RDI-specific thresholds, and the present findings should not be construed as altering those guidelines. However, prospective validation of such a risk calculator in independent cohorts is essential before it can guide routine clinical decisions such as primary antifungal prophylaxis or intentional RDI modification. In the interim, our results support implementing more vigilant infection monitoring and prevention strategies for patients receiving high-intensity chemotherapy, especially during periods of profound neutropenia.

## Data Availability

The data analyzed in this study is subject to the following licenses/restrictions: due to the inclusion of sensitive patient data that could potentially compromise personal identity information, and in accordance with the regulations of the Ethics Committee and the informed consent terms, the original dataset is not publicly available. Requests to access these datasets should be directed to QY; youqinghai@ahmu.edu.cn.
