# Supplementary material for: Influence of chemotherapy intensity on opportunistic fungal infection risk in non-small cell lung carcinoma: a retrospective study
Source: Front Med (Lausanne). 2026 Mar 30;13:1800575. doi: 10.3389/fmed.2026.1800575 (PMC13070931; doi:10.3389/fmed.2026.1800575)
Supplement: Supplementary file 1 [file Table_1.docx]

Supplementary Table S1. Multivariable Logistic Regression Model for Predicting Invasive Fungal Infection

| Variable | Coefficient | Standard Error | Odds Ratio  (95% CI) | P-value |
| --- | --- | --- | --- | --- |
| RDI (per 1% increase) | 0.052 | 0.018 | 1.053 (1.017-1.091) | 0.004 |
| Age (per year) | 0.021 | 0.015 | 1.021 (0.992-1.052) | 0.162 |
| Baseline Albumin (per g/L) | -0.114 | 0.041 | 0.892 (0.823-0.967) | 0.006 |
| Diabetes Mellitus (Yes) | 0.891 | 0.396 | 2.438 (1.122-5.297) | 0.024 |
| Intercept | -2.847 | 1.524 | - | 0.062 |
